# Simultaneous quantification of pyrethroid metabolites in urine of non-toilet-trained children in Japan

**DOI:** 10.1265/ehpm.21-00037

**Published:** 2022-06-14

**Authors:** Jun Ueyama, Yuki Ito, Risa Hamada, Naoko Oya, Sayaka Kato, Taro Matsuki, Hazuki Tamada, Kayo Kaneko, Shinji Saitoh, Mayumi Sugiura-Ogasawara, Takeshi Ebara, Michihiro Kamijima

**Affiliations:** 1 Department of Biomolecular Sciences, Field of Omics Health Sciences, Nagoya University Graduate School of Medicine, Nagoya 461-8673, Japan; 2 Department of Occupational and Environmental Health, Nagoya City University, Graduate School of Medical Sciences, Nagoya 467-8601, Japan; 3 Department of Pediatrics and Neonatology, Nagoya City University, Graduate School of Medical Sciences, Nagoya 467-8601, Japan; 4 Department of Obstetrics and Gynecology, Nagoya City University, Graduate School of Medical Sciences, Nagoya 467-8601, Japan

**Keywords:** Pyrethroid, Small children, Urine samples, Human biomonitoring

## Abstract

**Background:**

Pyrethroid (PYR) insecticides are widely used for controlling various pests. There are two types that differ in terms of usage: agricultural-purpose PYR (agriculture-PYR) and hygiene purpose PYR (hygiene-PYRs). Few studies exist on the exposure to these chemicals in small children. In this study, we conducted biomonitoring of urinary pyrethroid metabolites in 1.5-year-old children throughout the year.

**Methods:**

Study subjects were 1075 children participating in an Aichi regional sub-cohort of the Japan Environment and Children’s Study as of 18-month health check-up. The concentrations of four specific hygiene-PYR metabolites including 2,3,5,6-tetrafluoro-1,4-benzenedimethanol (HOCH_2_-FB-Al), and five common metabolites of hygiene- and agriculture-PYRs including 3-phenoxybenzoic acid (3PBA) and *cis*- and *trans*-3-(2,2-dichlorovinyl)-2,2-dimethylcyclopropane-1-carboxylic acid (DCCA), were measured in urine samples extracted from soiled diapers using a triple quadrupole gas chromatograph-mass spectrometer.

**Results:**

The highest detection frequencies were for 3PBA, followed by DCCA, 1R-*trans*-chrysanthemum dicarboxylic acid, and HOCH_2_-FB-Al. Among the six metabolites, urinary concentrations were seasonally varied. However, this variation was not observed in the most studied PYR metabolite, 3PBA. Spearman’s correlation analysis demonstrated a significant positive correlation between FB-Al and DCCA (*r* = 0.56) and HOCH_2_-FB-Al and 4-methoxymethyl-2,3,5,6-tetrafluorobenzyl alcohol (*r* = 0.60).

**Conclusions:**

This biomonitoring survey found widespread and seasonally specific exposure to multiple hygiene- and agriculture-PYRs in 1.5-year-old Japanese children.

## Background

Pyrethroids (PYRs) are synthetic neurotoxic insecticides derived from pyrethrum, a naturally occurring compound found in chrysanthemum flowers. PYRs are often used in croplands (hereafter agriculture-PYR), but are also used as household pesticides for controlling insects in living spaces, pets, and livestock as a spatial repellent (hereafter hygiene-PYR), because of their relative safety for humans and an immediate insecticidal effect with broad-range spectrum [1, 2]. In Japan, agriculture-PYRs are registered under the Agricultural Chemicals Regulation Act, but hygiene-PYRs are not under the act. The categorization of PYRs according to the registry basis is useful in terms of exposure control in human population when a risk management measure is required. While the amount of active ingredients of agriculture-PYR shipped throughout Japan decreased from 304 tons in 2,000 to 162 tons in 2018 [3], limited information exists on the hygiene-PYR consumption. The general population might be exposed to agricultural-PYRs via food and/or agriculture-related occupations, and to hygiene-PYRs mainly through daily activities, except for food consumption.

In recent decades, new knowledge has emerged about the special vulnerability of children to chemical exposure in their living space [4, 5], and their exposure to insecticides has also attracted attention internationally. The ubiquity of daily use of PYR and the perspective provided by some reports suggesting possible PYR-related adverse health effects on neurodevelopment in children has caused concern [6, 7]. Human biomonitoring (HBM) using urine, which can be conducted by non-invasive sampling, addresses the exposure level of chemicals through all exposure routes (dermal, gastrointestinal, and inhalation) in daily life. Although developmental toxicity essentially refers to adverse effects induced before and/or after birth, most HBM research aimed at children’s health has been performed in pregnant women. Therefore, comprehensive exposure assessment of PYRs in children is essential, particular in small children during a critical period of neurodevelopment after birth. However, information about PYR exposure level and its characteristics, such as concentration distribution and seasonal variation, is limited because of the difficulty in collecting specimens from small children.

Sensitive HBM of PYRs in human urine has been developed, largely because urine as a specimen presents obvious benefits in terms of the ease of sample collection from a large number of volunteers (especially small children) relative to blood samples. In epidemiological studies, urinary PYR metabolites, such as 3-phenoxybenzoic acid (3PBA), have been used as the most sensitive biomarkers for environmental PYR exposure since the late 1990s. 2,2-(Dichlorovinyl)-2,2-dimethylcyclopropane carboxylic acids (DCCA), 4-fluoro-3-phenoxybenzoic acid (FPBA), and *cis*-2,2-(dibromovinyl)-2,2-dimethylcyclopropane carboxylic acid (DBCA) have also been detected in urine samples from occupational and environmental epidemiology [8, 9]. In addition to introducing these PYR exposure markers, a sensitive and high-throughput determination method of urinary hygiene-PYR metabolites using a triple quadrupole gas chromatograph-mass spectrometer (GC-MS/MS) has been developed and successfully applied to quantify hygiene-PYR metabolites in urine samples of children [10]. Table 1 summarizes the PYR exposure markers, selected pesticides, and their uses. Moreover, this method provided opportunities for tracking exposure trends, which are rising in Japan [8]. Further studies are required for gathering fundamental scientific evidence related to the exposure of small children to PYRs using HBM.

**Table 1:** Pyrethroid (PYR) exposure markers, selected pesticides, and the uses.

**Exposure markers in urine** **(abbreviation)**	**Pyrethroids** **(examples)**	**Agricultural use**	**Hygienic use**
3-phenoxybenzoic acid (3PBA)	Cyhalothrin	X	
	Cypermethrin	X	
	Esfenvalerate	X	
	Fenpropathrin	X	
	Ethofenprox	X	
	Permethrin	X	X
	Phenothrin		X
	Tralomethrin	X	

4-fluoro-3-phenoxybenzoic acid (FPBA)	Cyfluthrin	X	

1R-*trans*-chrysanthemum dicarboxylic acid (CDCA)	Allethrin	X	X
	Imiprothrin		X
	Tetramethrin		X
	Prallethrin		X
	Resmethrin		X

*cis*-3-(2,2-dibromovinyl)-2,2-dimethylcyclopropane-1-carboxylic acid (DBCA)	Tralomethrin	X	

*cis*- and *trans*-3-(2,2-dichlorovinyl)-2,2-dimethylcyclopropane-1-carboxylic acid (DCCA)	Transfluthrin		X
	Permethrin	X	X
	Cypermethrin	X	
	Cyfluthrin	X	

4-methyl-2,3,5,6-tetrafluorobenzyl alcohol (CH_3_-FB-Al)	Profluthrin		X

4-methoxymethyl-2,3,5,6-tetrafluorobenzyl alcohol (CH_3_OCH_2_-FB-Al)	Metofluthrin		X

2,3,5,6-tetrafluorobenzyl alcohol (FB-Al)	Transfluthrin		X

2,3,5,6-tetrafluoro-1,4-benzenedimethanol (HOCH_2_-FB-Al)	Metofluthrin		X
	Profluthrin		X

We developed a quantitative method to measure 3PBA and DCCA of diaper-absorbed urine samples [11], indicating that this method can evaluate PYR exposure levels in small children without any drawbacks related to the use of urine collection packs. Although it was necessary to clarify the exposure levels occurring during the critical period of neurodevelopment after birth, limited information about urinary concentrations of agriculture-PYR metabolites and no information about those of hygiene-PYR metabolites in small children exist. This study aimed to clarify the concentration distribution, its seasonal differences, and correlations between PYR metabolites in urine in 1.5-year-old Japanese children using diapers.

## Methods

### Study subjects and design

The present study was conducted as an adjunct study of Japan Environment and Children’s Study (JECS), an ongoing prospective nationwide birth cohort study in Japan. The main aim of the JECS was to investigate associations of environmental factors with children’s health and development [12]. Ethics committees of Nagoya University Graduate School of Medicine and Nagoya City University Graduate School of Medical Sciences approved this adjunct study protocol. The JECS main study protocol was approved by the Ministry of the Environment, Japan.

The present study population was the JECS participants who were registered in the Aichi regional sub-cohort of the JECS (JECS-A). In JECS-A, 5721 pregnant women living in Ichinomiya City and Nagoya City, Japan, and their 5554 children were included [13]. A part of these children, who attended a municipal health check program covering 18-month children, were recruited for this study. A total of 1077 participants (16–23 months old (mean, 18.7 months old) were enrolled (representing 77.9% of solicited respondents)). The study has been designed to cause no pain to participants and does not require a complex protocol. However, the maternal age (margin of average was 0.5 years), passive smoking opportunities, and paternal academic background were statistically different between the participants of this study and the other participants in JECS-A. Soiled diapers or morning-void urine samples were collected from children between June 8, 2015, and August 19, 2016 (first morning-void urine was collected from one child). One participant withdrew consent and another provided less urine, which was insufficient for the measurement. Thus, a total of 1075 samples were provided for CDCA, DCCA, DBCA, FPBA, and 3PBA assay. The monthly numbers of subjects from January to December were as follows: 97, 119, 105, 51, 48, 95, 94, 106, 106, 88, 79, and 87. The percentage range of boys in each month was between 39% (June) and 58% (August).

### Standard PYR metabolites and internal standards

The urinary PYR metabolites we assayed are summarized in Table 1. 3PBA (purity 99%) and the internal standard (I.S.) 2-phenoxybenzoicacid (purity 98%) were purchased from FUJIFILM Wako Pure Chemical Industries, Ltd. (Osaka, Japan). Isotope-labeled HOCH_2_-FB-Al (purity 99.9%) was used as an I.S., and *trans*-CDCA (purity 98.9%) were custom-synthesized at Sumika Technoservice Corporation (Hyogo, Japan). DBCA was purchased from Chem Service, Inc. (West Chester, PA, USA). 2,3,5,6-Tetrafluorobenzyl alcohol (FB-Al) (purity >96.0%) and HOCH_2_-FB-Al (purity >97.0%) were obtained from Tokyo Chemical Industry (Tokyo, Japan). FPBA and *cis*- and *trans*-DCCA (purity 98%) were purchased from Cambridge Isotope Laboratories (Tewksbury, MA, USA). CH_3_OCH_2_-FB-Al (>97.0%) and CH_3_-FB-Al (purity 99.8%) were purchased from Tokyo Kasei Kogyo (Tokyo, Japan).

### Determination of urinary metabolites

Soiled diapers were collected from children in the morning and transferred to the laboratory in refrigerated cargo the following day. Urine was extracted immediately upon arrival of the diaper as described in our previous report [14]. Briefly, the urine absorber containing urine was removed from the diaper into a 10-mL syringe. This syringe and a 20-mL syringe containing acetone were connected, and the acetone was manually reciprocated between the syringes five times to extract the urine from the absorber. The eluate was then poured into a glass test tube. The urine absorber was dried in a vacuum state for approximately 2 h. The extracts containing acetone were evaporated at 40 °C on a heat block with a gentle nitrogen stream for obtaining the urine. The extracted urine samples were stored at −80 °C until analysis.

The CDCA, DCCA, DBCA, FPBA, and 3PBA measurements were applied to all samples, while CH_3_-FB-Al, CH_3_OCH_2_-FB-Al, FB-Al, and HOCH_2_-FB-Al were assayed for randomly selected samples (n = 780). That was because of the constraints of cost and labor that were necessary to measure the metabolites in all the collected samples. The monthly number of subjects from January to December was as follows: 78, 88, 72, 39, 34, 67, 43, 83, 84, 62, 57, and 73. The percentage of boys was between 42 (February) and 54 (November).

Using an Agilent 7890A GC equipped with an Agilent 7000B GC-MS/MS system (Agilent, Inc., CO, USA), the urinary PYR metabolites were determined according to the procedure reported by Ueda et al. [10]. The limits of detection (LODs) were determined as concentrations with signal-to-noise ratios of 3. Analyte LODs were as follows: 0.10 for FB-Al, 0.24 for CH_3_-FB-Al, 0.15 for CH_3_OCH_2_-FB-Al, and 0.04 µg/L for other analytes. Quality control (QC) samples were analyzed every 40 samples measurement. The highest coefficient variation was observed for FB-Al (14.6%). The absolute recoveries of PYR metabolites from diapers were 68% for CDCA, 77% for DCCA, 81% for DBCA, 84% for FPBA, 79% for 3PBA, 59% for FB-Al, 53% for CH_3_-FB-Al, 71% for CH_3_OCH_2_-FB-Al, and 78% for HOCH_2_-FB-Al. These values were used as adjustment factors to correct for analyte loss from diapers to correct urinary PYR concentrations. No target analytes were detected from blank diapers.

### Statistical analysis

Urinary concentrations of PYR metabolites are summarized and presented using statistical characteristics (sample size, number of detection frequency, geometric mean (GM), 95% confidence interval for the GM, selected percentiles, and maximum value) for the total sample. When the analysis of CDCA, DCCA, DBCA, FPBA, and 3PBA, all measurement data has been included. On the other hand, when the analysis of FB-Al, CH_3_-FB-Al, CH_3_OCH_2_-FB-Al, HOCH_2_-FB-Al, samples that did not measure them were excluded. Undetectable urinary concentrations of analytes were assigned to an LOD value divided by the square root of 2 [15]. Differences in urinary metabolite concentrations during hot season (from June to September) were tested using the Mann-Whitney *U* test. Spearman’s correlation coefficient was calculated for determining the relationship between each biomarker concentration with logarithmic conversion. Moreover, Spearman’s correlation was also calculated by excluding <LOD samples. Samples that were not measured for CH_3_-FB-Al, CH_3_OCH_2_-FB-Al, FB-Al, and HOCH_2_-FB-Al were also excluded from the correlation tests. Statistical significance was defined as *p* < 0.01. All statistical analyses were performed using the statistical software JMP® 13 (SAS Institute Inc., Cary, NC, USA).

Urinary creatinine (Cre) concentrations were measured using LC-MS/MS, as described previously [14]. Cre was also corrected by the rate of absorption of a diaper. The regression equation was y = 0.9964x + 0.0694, and *R*^2^ was 1.00.

## Results

Table 2 summarizes the descriptive statistics for the urinary PYR metabolites. Volume-based concentrations (µg/L) and Cre-corrected concentrations (µg/g Cre) of PYR metabolites are presented. The metabolites 3PBA, DCCA, and CDCA were detected at high frequencies in 98%, 96%, and 57% of urine samples, respectively. Lower frequencies were observed for FPBA, DBCA, CH_3_-FB-Al, CH_3_OCH_2_-FB-Al, FB-Al, and HOCH_2_-FB-Al (less than 36%). The median and 95th percentile for 3PBA, CDCA, and DCCA were 0.98 and 4.89, 0.09 and 1.56, and 0.51 and 10.98 µg/L, respectively.

**Table 2:** Summary of pyrethroid (PYR) metabolite concentrations in diapered children urine.

	**Analytes**	**>LOD** **(%)^a^**	**GM**	**Min.**	**Selected percentile**					**Max.**	**95%CI median**
					**5th**	**25th**	**50th**	**75th**	**95th**		
µg/L											
	3PBA	98	1.11	<LOD	0.42	0.71	0.98	1.56	4.86	28.20	0.95–1.02
	FPBA	12	-^b^	<LOD	<LOD	<LOD	<LOD	<LOD	0.09	1.62	-^b^
	CDCA	57	-^b^	<LOD	<LOD	<LOD	0.09	0.33	1.56	22.87	0.07–0.10
	DBCA	22	-^b^	<LOD	<LOD	<LOD	<LOD	<LOD	0.17	2.17	-^b^
	DCCA	96	0.59	<LOD	0.06	0.18	0.51	1.54	10.98	121.34	0.46–0.59
	CH_3_-FB-Al	10	-^b^	<LOD	<LOD	<LOD	<LOD	<LOD	0.75	20.66	-^b^
	CH_3_OCH_2_-FB-Al	18	-^b^	<LOD	<LOD	<LOD	<LOD	<LOD	0.73	4.96	-^b^
	FB-Al	33	-^b^	<LOD	<LOD	<LOD	<LOD	0.18	0.67	14.36	-^b^
	HOCH_2_-FB-Al	36	-^b^	<LOD	<LOD	<LOD	<LOD	0.09	0.50	5.46	-^b^
µg/g Cre											
	3PBA		2.25	<LOD	0.76	1.39	2.07	3.35	10.01	52.17	1.98–2.16
	FPBA		-^b^	<LOD	<LOD	<LOD	<LOD	<LOD	2.88	3.16	-^b^
	CDCA		-^b^	<LOD	<LOD	<LOD	0.15	0.66	3.10	243.06	0.12–0.19
	DBCA		-^b^	<LOD	<LOD	<LOD	<LOD	<LOD	0.24	4.89	-^b^
	DCCA		1.15	<LOD	0.14	0.42	1.08	2.88	18.91	226.65	0.97–1.21
	CH_3_-FB-Al		-^b^	<LOD	<LOD	<LOD	<LOD	<LOD	1.68	28.93	-^b^
	CH_3_OCH_2_-FB-Al		-^b^	<LOD	<LOD	<LOD	<LOD	<LOD	1.24	8.19	-^b^
	FB-Al		-^b^	<LOD	<LOD	<LOD	<LOD	0.35	1.40	27.71	-^b^
	HOCH_2_-FB-Al		-^b^	<LOD	<LOD	<LOD	<LOD	0.19	0.94	4.84	-^b^

*LOD* limit of detection, *GM* geometric mean, *95%CI median* approximate 95% confidence interval for the median value

^a^Percent of detection frequency

^b^GM and 95%CI median was not calculated due to low detection rate

**Fig. 1:**
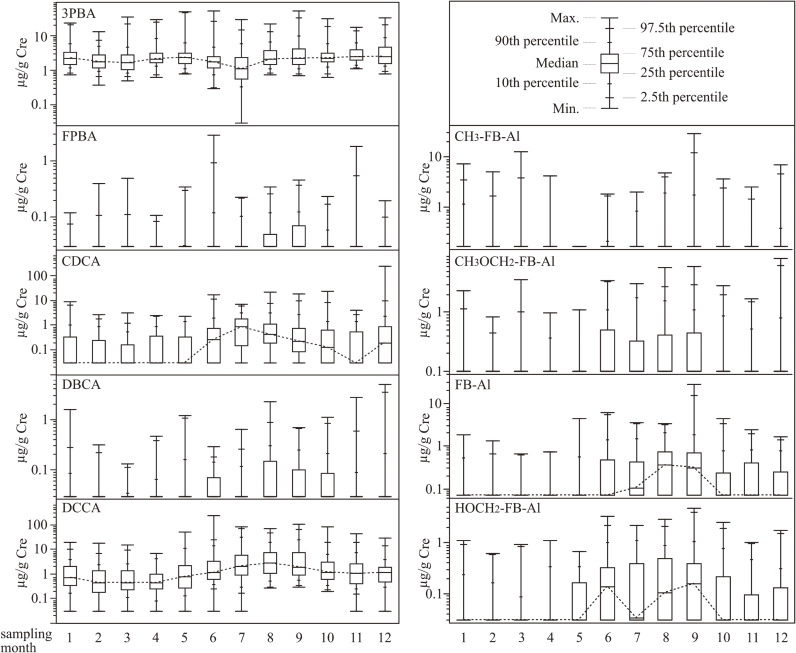
Boxplot of urinary concentration of pyrethroid (PYR) metabolites (µg/g Cre) throughout a year. The dotted line connects the median values of each month. The limits of detection (LOD) values divided by the square root of 2 were assigned to urine samples with undetectable PYR metabolites. The value in parentheses below the x-axis indicates the number of subjects in each month.

The boxplot in Fig. 1 represents the monthly data of PYR metabolite concentrations for 12 months, depicting data distribution with 7 values including 5 percentile values and minimum and maximum values, as shown in the upper right panel. Figure 1 shows the non-parametric distribution of concentrations. Notably, 75th percentile or median values of PYR metabolite concentrations were higher from June to September, in which the average temperature was highest in the year (hereafter called the hot season) (Japan Meteorological Agency) [16]. The amount of PYR metabolites in urine during the hot season was significantly higher than that in other seasons, except for 3PBA (*p* < 0.001, Mann-Whitney *U* test). No obvious features were observed for any of the PYR metabolite concentrations during other seasons.

**Fig. 2:**
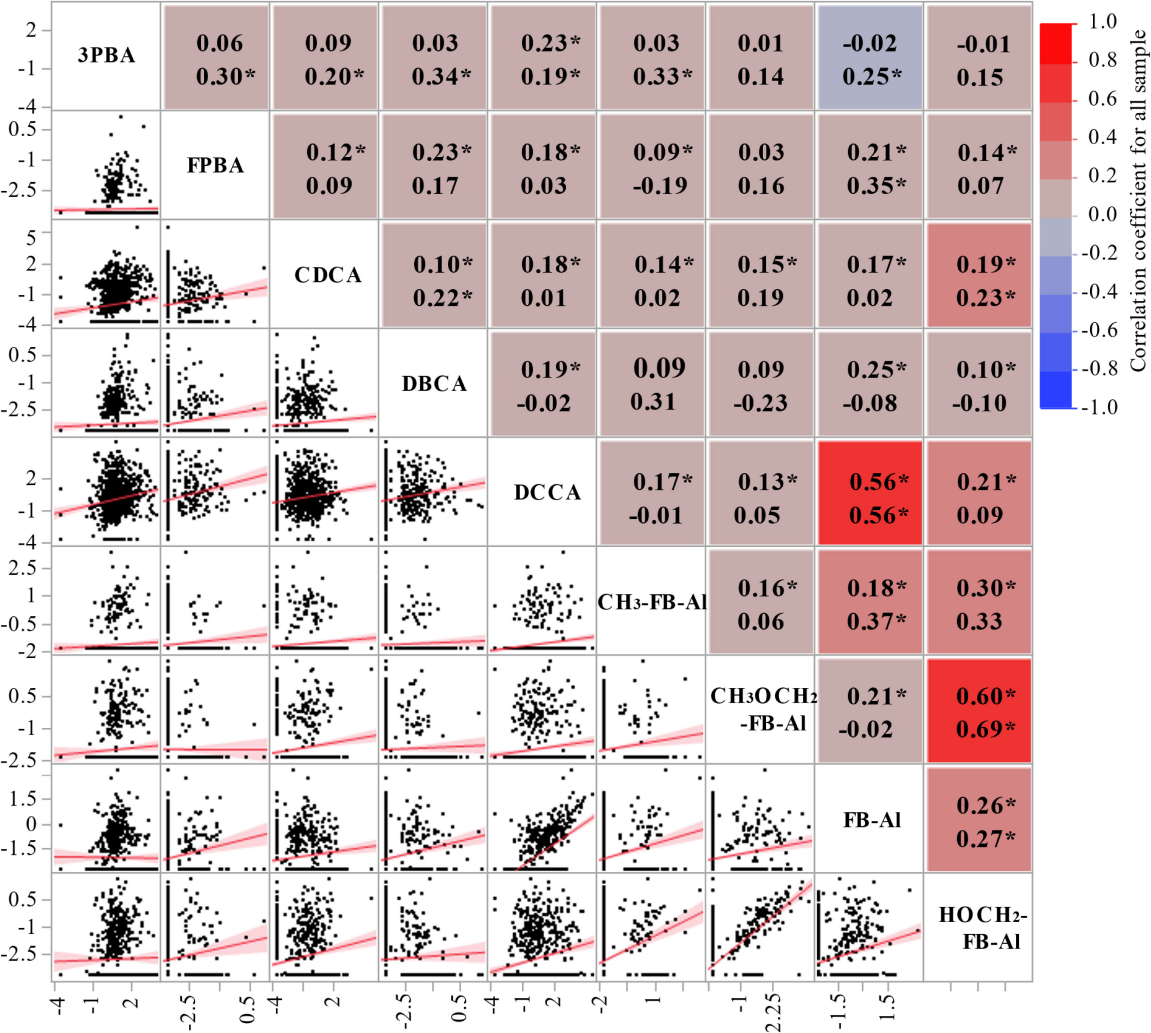
Scatter plots and coefficients of correlations between each log-transformed concentration of biomarkers (µg/g Cre). The value indicates coefficients of correlations for all sample analysis (upper) and without under LOD samples (in parentheses). Asterisks indicate a *p* value less than 0.01.

The associations between urinary PYR concentrations are illustrated in Fig. 2 for the 1.5-year-old male and female study participants. Moderate correlations, that is, correlation coefficients greater than 0.5, were observed between DCCA and FB-Al (Spearman’s correlation coefficient (*r*) = 0.56, *p* < 0.01) and between CH_3_OCH_2_-FB-Al and HOCH_2_-FB-Al (*r* = 0.60, *p* < 0.01). Although significant correlations were detected between urinary 3PBA, FPBA, and DBCA and some PYR metabolites, these correlation coefficients were extremely low.

## Discussion

Small children are known to be vulnerable to toxic chemicals. This study yielded three significant findings. First, the 9 urinary PYR metabolites in a large number of 1.5-year-old children were successfully quantified, and the concentration distribution was presented. These results are the first to demonstrate that many small children are exposed to agriculture- and hygiene-PYR at no acute toxic levels in their daily lives. Second, there were seasonal differences in concentrations of some urinary PYR metabolites. Eight of the nine assessed biomarkers were higher in the hot season in Japan. This indicates that the PYR exposure assessment using biomonitoring techniques should consider the sampling season and PYR exposure markers that should be monitored. Third, we found positive associations between some urinary hygiene-PYR metabolites. This result suggests that daily co-exposure to a part of hygiene-PYR may occur in Japanese small children.

Some biomonitoring studies on urinary PYR metabolites in pregnant women in Mexico and France [17, 18], children aged 3 to 14 years in Germany [19], and young- and middle-aged adults in the U.S [20] have been reported. In comparison with these previous studies, the strengths of the present study are the large number of small children as study subjects and PYR metabolite monitoring. The present study revealed that many diapered children were ubiquitously exposed to agriculture- and hygiene-PYRs in a wide concentration range. We have reported urinary PYR concentrations in approximately 200 3-year-old Japanese children, which showed median values (µg/L and µg/g Cre) of 1.01 and 1.40 for 3PBA, 0.14 and 0.18 for CDCA, and 0.72 and 1.00 for DCCA [21]. These results were approximately the same as the concentrations observed in the present study. Most PYRs exhibit short half-lives. Urine analysis in this study may have estimated recent exposure because most of the absorbed dose is eliminated by the kidneys within a few days [22]. The human biomonitoring reference values (RV95), which are defined as the 95^th^ percentile of the measured pollutant concentration levels in the relevant matrix of the reference population for 3PBA and DCCA have been established in Germany for 3–14 years of age [23] and Canada for 3–79 years of age [24]. RV95 in Germany and Canada are 2 and 5.7 µg/L for 3PBA and 3 (sum of *cis* and *trans* form) and 0.39 µg/L (*cis* form) for DCCA, respectively. The 95^th^ percentile value of 4.86 µg/L of 3PBA in this study was approximately identical to that of Canadian RV95. Conversely, the 95^th^ percentile value of 10.98 µg/L of DCCA (sum of *cis* and *trans* form) in this study was about four-fold that of Germany, indicating that Japanese children may have higher exposure to transfluthrin, permethrin, cypermethrin, and/or cyfluthrin than in Germany and Canada. There are some limitations to these comparisons because of the age differences between the subjects that were used for RV95 development and the small children in the present study. More recently, Yoshida et al. reported urinary PYR metabolites in 132 Japanese children (6–15 years old), showing a median concentration of 0.58 µg/g Cre for 3PBA and 0.74 µg/g Cre for DCCA [25]. However, it is important to be careful when comparing urinary PYR metabolite levels between different ages. Because, urine volume and the Cre concentration of 24 h urine change with age, especially in small children [26, 27]. Notably, a large proportion of small children in Japan are exposed to PYR at environmental background levels in their daily lives.

Of the few reports on seasonal-difference in PYR exposure, Klimowska et al. [28] assessed the urinary excretion of 3PBA, DBCA, DCCA, and FPBA in monthly 24-h urine samples from children (n = 2) and adults (n = 12). According to the study, there were no significant differences in the concentration of metabolites in urine samples collected at different times of the year. On the other hand, Lu et al. [29] reported that the concentration of 3PBA in children has a statistically significant seasonal difference. In our results, FPBA, DBCA, and DCCA appeared to be slightly higher during the hot season, although no obvious seasonal difference was found in the 3PBA concentration. The seasonal variation we observed may not have been caused by individual differences such as selection bias, because urinary metabolite levels tend to increase and then decrease during the hot season. 3PBA is a common metabolite excreted after exposure to agriculture-PYR and hygiene-PYR (Table 1). Recently, Rodzaj et al. [30] reported that non-dietary factors, especially dog ownership and pesticide use in household pets, significantly contribute to urinary 3PBA concentrations. Moreover, several dietary sources of PYR have been indicated, including grain products, such as bread, and margarine. These factors do not have obvious seasonal variations and depend on the usage or consumption situation in different countries and may have led to the result that there is no seasonal variation in urinary 3PBA. The yearly average amount of expenditure per household (two-or-more-person households) of insecticide and moth repellent is approximately 21 USD (2,400 JPY) in the last decade [31]. Approximately 60% of them are paid in the Japanese hot season, indicating that the exposure levels of some hygiene-PYR exposure are expected to be higher than in the other seasons [31]. Most of all urinary metabolite levels during the hot season were also likely to be higher than in the other seasons. Of these, CH_3_OCH_2_-FB-Al and FB-Al are specific metabolites of metofluthrin and transfluthrin, respectively. Thus, the exposure level of metofluthrin and transfluthrin during summer may be higher than during other seasons. One interesting finding was that a small number of children were exposed to hygiene-PYR exposure during the winter season. This may be due to either the use of PYR products, even in the winter or exposure to residual PYRs used during summer. The present results indicate that the season of sampling is an important factor for reliable exposure assessment when further studying the health risks of PYR using HBM of PYR metabolites with seasonal variation. Further studies are needed to establish sampling methods for reliable chronic PYR exposure assessments. Moreover, further studies are needed for clarifying the exposure route (e.g., inhalation, dermal absorption, or oral exposure by hand-to-mouth behavior and house dust intake) of hygiene-PYRs because of the wide variety of products and applications.

The correlation between urinary metabolite concentrations may have resulted from co-exposure or common metabolites among the PYRs. The apparent positive relationship between urinary levels of DCCA and FB-Al, but not 3PBA, suggests that the major source of DCCA in children’s urine might have occurred as a result of exposure to transfluthrin. The concentration of HOCH_2_-FB-Al (a specific metabolite of metofluthrin) was associated with CH_3_OCH_2_-FB-Al (a common metabolite of metofluthrin and profluthrin) (*r* = 0.67). On the other hand, a very low correlation coefficient was observed between HOCH_2_-FB-Al and CH_3_-FB-Al (a specific metabolite of profluthrin). Since the specific metabolite of metofluthrin is HOCH_2_-FB-Al, it is surely probable that the major exposure origin of CH_3_OCH_2_-FB-Al is metofluthrin, but not profluthrin. No clear correlations were found between 3PBA and other metabolites, partly because 3PBA is a common metabolite in many PYRs.

## Strengths, limitations, and future perspective

The strengths of this study include data from a relatively large cohort of small children and the HBM data of multiple PYR metabolites in urine. Several limitations should be considered when interpreting our results. First, only morning void urine was measured as a representative individual sample. Secondly, although there were probably minor selection biases that might not have a significant impact on the results of HBM, these data do not constitute random probability samples of all Japanese children, so there may have been some potential selection bias in the results. This selection bias restricts the generalization of these results to the entire population of small children in Japan. Third, the detection frequency of some urinary metabolite in this study were less than 50%, and thus a more sensitive analysis might provide more insight into the PYR exposure. Finally, HBM studies for PYR exposure assessment will become more important as the demand for spatial use of repellents may increase in the future.

## Conclusion

It was found that 1.5-year-old Japanese children continue to be exposed to agriculture-PYRs throughout the year and are temporarily exposed to hygiene-PYRs (especially during summer). Given these findings, scientists should be cautious when selecting urine sampling time points and determining biomarkers for PYR exposure in future exposure and environmental epidemiology studies.

## Abbreviations

CDCA: 1R-*trans*-chrysanthemum dicarboxylic acid
CH_3_-FB-Al: 4-methyl-2,3,5,6-tetrafluorobenzyl alcohol
CH_3_OCH_2_-FB-Al: 4-methoxymethyl-2,3,5,6-tetrafluorobenzyl alcohol
Cre: creatinine
DBCA: *cis*-2,2-(dibromovinyl)-2,2-dimethylcyclopropane carboxylic acid
DCCA: *cis*- and *trans*-3-(2,2-dichlorovinyl)-2,2-dimethylcyclopropane-1-carboxylic acid
FB-Al: 2,3,5,6-tetrafluorobenzyl alcohol
FPBA: 4-fluoro-3-phenoxybenzoic acid
GC-MS/MS: Triple quadrupole gas chromatograph-mass spectrometer
GM: Geometric mean
HBM: Human biomonitoring
HOCH_2_-FB-Al: 2,3,5,6-tetrafluoro-1,4-benzenedimethanol
I.S.: Internal standard
JECS: Japan Environment and Children’s Study
LC-MS/MS: high-performance liquid chromatography with tandem mass spectrometry
LOD: Limits of detection
PYR: Pyrethroid
QC: Quality control
3PBA: 3-phenoxybenzoic acid

## Declarations

### Availability of data and materials

The data and materials used to derive our conclusions are unsuitable for public deposition because of ethical restrictions and the specific legal framework in Japan. It is prohibited by the Act on the Protection of Personal Information (Act No. 57 of May 30, 2003, amended on September 9, 2015) to publicly deposit data containing personal information. The Ethical Guidelines for Epidemiological Research enforced by the Japan Ministry of Education, Culture, Sports, Science and Technology and the Ministry of Health, Labor and Welfare also restrict the open sharing of epidemiologic data.
